# Early identification of monogenic diabetes: implications on medical treatment and genetic counselling for an adolescent girl with MODY3

**DOI:** 10.1186/1687-9856-2015-S1-P19

**Published:** 2015-04-28

**Authors:** Wai-chun Wong, Chi-tak Tong

**Affiliations:** 1 Department of Paediatrics & Adolescent Medicine, Alice Ho Miu Ling Nethersole Hospital, Hong Kong, China

## Aim

To highlight the therapeutic and genetic implications of early diagnosis of HNF-1α mutation in a girl whose family member had previously been thought to have type 1 diabetes.

## Method

We reported an adolescent girl in whom a diagnosis of MODY3 was established by molecular finding of a heterozygous HNF-1α NM 000545.5: c.775G>T (p.Val259Phe) mutation. She and her mother now successfully manage their diabetes with low dose sulphonylurea.

## Result

A 14 years old girl, who previously had been well, was incidentally found to have hyperglycemia by her diabetic mother. Her body mass index was 19 kg/m^2^. General examination was unremarkable without any acanthosis nigricans. Fasting blood glucose was 6.4mmol/L and 2 hour glucose in oral glucose tolerance test was 15.3mmol/L. HbA1c was 7.5%. Fasting C-peptide was 3.2 µg/L (reference 0.9-7.1µg/L).

Family history revealed that her mother developed diabetes at the age of 20 and had been managed as type 1 diabetes with insulin. Mother had satisfactory glycemic control despite the dosage of insulin remained relatively low for decade. However, she then got poor compliance to insulin injection after the age of 40, resulted in worsening of HbA1c to 11-12%. Furthermore, two maternal aunts were thought to have diabetes, but no further detail was available.

A diagnosis of MODY was considered for the girl and molecular analysis confirmed MODY3 with heterozygous HNF-1α NM 000545.5: c.775G>T (p.Val259Phe) mutation. The patient was initiated on the treatment with low dose gliclazide. She could achieve good glycemic control without problem of significant hypoglycemia. Her most recent HbA1c was 5.7%.

Gene sequencing confirmed that her mother also harbored the same mutation. After genetic counselling, insulin therapy of patient’s mother was gradually switched to glimepiride by adult endocrinologist. Her HbA1c showed significant improvement from 12.2% to 7.1%. In addition, no mutation was detected for patient’s elder sister, indicated that she only had the population risk of developing diabetes.

## Conclusion

Maturity Onset Diabetes of the Young (MODY) is frequently misdiagnosed as type 1 or type 2 diabetes. MODY3, resulting from a mutation in the HNF-1α gene, is the most common form of MODY and low dose sulphonylurea is highly effective in maintaining a good glycemic control. Early awareness of MODY and confirmation of subtypes by molecular analysis is important in guiding the appropriate treatment, predicting the clinical course, providing genetic counselling and sometimes, correcting the diagnosis or treatment for other diabetic family members.

Written informed consent was obtained from the patient for publication of this abstract and any accompanying images. A copy of the written consent is available for review by the Editor of this journal.

